# Recovery of Latent HIV-1 from Brain Tissue by Adoptive Cell Transfer in Virally Suppressed Humanized Mice

**DOI:** 10.1007/s11481-021-10011-w

**Published:** 2021-09-15

**Authors:** Hang Su, Sruthi Sravanam, Brady Sillman, Emiko Waight, Edward Makarov, Saumi Mathews, Larisa Y. Poluektova, Santhi Gorantla, Howard E. Gendelman, Prasanta K. Dash

**Affiliations:** 1 Department of Pharmacology and Experimental Neuroscience, College of Medicine, University of Nebraska Medical Center, Omaha, NE, United States; 2 Department of Pharmaceutical Sciences, College of Pharmacy, University of Nebraska Medical Center, Omaha, NE, United States

**Keywords:** Human immunodeficiency virus type one (HIV-1), humanized mice, adoptive transfer, brain, latent virus recovery, mouse viral outgrowth assay

## Abstract

Defining the latent human immunodeficiency virus type 1 (HIV-1) burden in the human brain during progressive infection is limited by sample access. Human hematopoietic stem cells (hu-HSCs)-reconstituted humanized mice provide an opportunity for this study. The model mimics, in measure, HIV-1 pathophysiology, transmission, treatment, and elimination in an infected human host. However, to date, brain HIV-1 latency in hu-HSC mice during suppressive antiretroviral therapy (ART) was not studied. To address this need, hu-HSC mice were administered long acting (LA) ART 14 days after HIV-1 infection was established. Animals were maintained under suppressive ART for 3 months, at which time HIV-1 infection was detected at low levels in brain tissue by droplet digital polymerase chain reaction (ddPCR) test on DNA. Notably, adoptive transfer of cells acquired from the hu-HSC mouse brains and placed into naive hu-HSC mice demonstrated viral recovery. These proof-of-concept results demonstrate replication-competent HIV-1 reservoir can be established in hu-HSC mouse brains that persists during long-term ART treatment. Hu-HSC mice-based mouse viral outgrowth assay (hu-MVOA) serves as a sensitive tool to interrogate latent HIV-1 brain reservoirs.

## Graphical Abstract

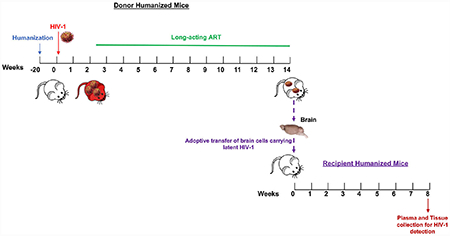

## Introduction

The elimination of the human immunodeficiency virus type 1 (HIV-1) from an infected human host remains a focus of world-wide research efforts. Indeed, virus remains integrated within the human genome during the life of the cell as transcriptionally silent. This precludes immune surveillance and viral clearance. Antiretroviral therapy (ART) suppresses viral growth but cannot eliminate infection. A primary challenge in attempts to eliminate HIV-1 rests in the broad distribution of latent infected cells throughout the body. While a majority of studies have focused on readily accessible peripheral blood mononuclear cells (PBMCs), virus is present in lymph nodes, gut-associated lymphoid tissue, bone marrow, spleen, the genitourinary system and the central nervous system (CNS). Each and all of these tissues are responsible for the persistent viral reservoir and for HIV-associated comorbidities (Lerner, et al. 2019). Amongst these tissue sites, the CNS serves as a notable HIV-1 anatomical site where viral suppression can be affected by inherent ART penetrance and migration of immune cells (Hong and Banks 2015; McGee, et al. 2006). As a result, HIV-1 persists and induces low-level inflammation which can herald HIV-associated neurocognitive disorders (HANDs) (Clifford and Ances 2013; Heaton, et al. 2010; Saylor, et al. 2016). While interrogating brain reservoirs is clinically challenging, autopsy examinations of individuals with premortem undetectable viremia identified HIV-1 brain DNA with as yet not fully determined viral replication-competence (Chaillon, et al. 2020; Lamers, et al. 2016). Thus, a recurrent challenge for HIV-1 latency studies depends on both detecting and measuring viral tissue reservoirs (Abdel-Mohsen, et al. 2020).

A limitation in detecting replication competent virus is that conventional qPCR amplifies both defective and replication-competent proviral DNA and results in overestimating the “true” reservoir size. While the standard viral outgrow assay (QVOA) readily detects virus from latent sites it fails to capture all (Ho, et al. 2013), especially in the face of a limited reservoir size (Henrich, et al. 2014; Luzuriaga, et al. 2015). Using QVOA as a standard, a mouse-based VOA (MVOA) was developed where peripheral blood mononuclear cells (PBMCs) from HIV-1 infected and ART suppressed persons were adoptively transferred into humanized mice (Metcalf Pate, et al. 2015). This assay enabled HIV-1 to be successfully recovered from the animals in the face of a negative QVOA (Charlins, et al. 2017). The results demonstrated that MVOA is a more sensitive assay exceeding QVOA in detecting replication-competent HIV-1. Recently, our group extended MVOA using humanized mice as donors and enabled a humanized mouse-to-mouse VOA (hu-MVOA) detection system. By adoptively transferring PBMCs from virally suppressed into naive humanized mice, HIV-1 recovery was made (Su, et al. 2020). In addition, these works allowed studies of human tissue cells that carry latent virus present in virally suppressed donor humanized mouse including splenocytes and bone marrow (BM) cells. These are major anatomical sites of latent HIV-1 supporting the fact that hu-MVOA can serve as a platform for detecting infected cells within a broad number of tissue reservoirs. Based on this notion in the current study, we tested whether adoptive transfer of brain cells from infected and ART suppressed humanized mice can be seen with high sensitivity. Donor humanized mice initiated long acting ART (Edagwa, et al. 2017) after 2 weeks of HIV-1 infection and were maintained under suppressive treatment for 3 months. Although only minimal HIV-1 infection was seen by ultrasensitive semi-nested ddPCR assay in donor humanized mouse brains before adoptive cell transfer, viral recovery was made from two of four recipient humanized mice. These results demonstrate that replication-competent HIV-1 persists in CNS during suppressive ART. Hu-MVOA provides a tool to evaluate HIV-1 CNS reservoirs.

## Materials and Methods

### Generation, HIV-1 Infection and Treatment of Humanized Mice

All experimental protocols were approved by the University of Nebraska Medical Center Institutional Animal Care and Use Committee (IACUC). All animal studies were performed in accordance with UNMC institutional policies and the National Institutes of Health guidelines. The experimental procedures were as follows. New-born NOD/scid-IL-2RγC^null^ (NSG) mice were injected with human CD34+ cord blood hematopoietic stem cells (hu-HSCs) through an intrahepatic route(Gorantla, et al. 2010b). Humanization was determined by flow cytometric staining of human cell markers. At 20-week age, donor hu-HSC mice with affirmed human cell counts (peripheral human CD45+ cells > 20%) were selected and intraperitoneally infected with HIV-1_ADA_ at a titer of 10^4^ tissue culture infection dose_50_ (TCID_50_)/animal. At 2 weeks following viral infection, plasma HIV-1 RNA was measured in blood samples recovered from the submandibular vein by automated COBAS Ampliprep System V2.0/Taqman-48 system assay (Roche Molecular Diagnostics, Basel, Switzerland). Donor hu-HSC mice with affirmed viral infection were initiated with a combination of first generation 4 ART regimens [LA dolutegravir (NMDTG), rilpivirine (NRPV), lamivudine (NM3TC), and abacavir (NMABC)] (Dash, et al. 2019). All the nanoformulations were given intramuscularly. NMDTG and NRPV were injected once every 4 weeks at doses equivalent to 45 mg/kg of parent drugs and NMABC and NM3TC were administered once a week at doses equivalent to 50 mg/kg of parent drugs. Donor hu-HSC mice were maintained on ART for an additional 12 weeks before sacrifice.

### Brain Adoptive Transfer

After 12 weeks of LA-ART treatment, plasma HIV-1 RNA of four donor hu-HSC mice was determined by automated COBAS Ampliprep System V2.0/Taqman-48 system and confirmed undetected (< 200 copies/ml). Animals were immediately sacrificed and brain cells were isolated and counted using the TC-20 automated cell counter (Bio-Rad, Hercules, CA). The whole cell population which contained both mouse and human cells were intraperitoneally injected into respective naive hu-HSC mice. The recipient hu-HSC mice were maintained for 8 more weeks to monitor HIV-1 propagation.

### Flow Cytometry

Hu-HSC mouse blood was collected to monitor human cell levels using monoclonal antibodies to human CD45, CD3, CD19, CD4, and CD8 (BD Pharmingen, San Diego, CA). Flow cytometry was operated on BD LSRII (BD Immunocytometry Systems, Mountain View, CA) system and data were analyzed using FlowJo software (BD Pharmingen, San Diego, CA).

### qPCR for HIV-1 DNA/RNA

Total DNA and RNA were extracted from hu-HSC mouse tissues including brain, lung, liver, spleen, bone marrow, gut, and kidney, by Qiagen All Prep DNA/RNA Mini Kit (QIAGEN, Hilden, Germany). RNA was first reverse transcribed to cDNA using Thermo-Fisher Scientific Verso cDNA Synthesis Kit (Invitrogen, MA). Semi-nested real-time PCR was performed to measure total HIV-1 DNA and RNA as previously described (Arainga, et al. 2016). In brief, the first round of PCR was performed on a conventional PCR machine (T100 Thermal Cycler, Bio-Rad, CA) in 25 μl of PCR reaction mix containing 500 ng of template and 50 ng each of both primers annealing to HIV-1 gag region and the reaction conditions are as follows: 94 °C for 3 min, followed by 15 cycles of 94 °C for 30s, 55 °C for 30s, and 72 °C for 1 min. The product of the first PCR was subsequently used as a template in the second semi-nested real-time qPCR amplification. The assay was performed on the ABI Step One Plus real-time PCR machine (Applied Biosystems, Foster City, CA) using TaqMan detection probe and primers. Five microliters of the first PCR product were diluted to 10 μl with PCR master mix containing two primers at 0.2 μM each and 0.2 μM TaqMan dual-labeled fluorescent probe. Real-time PCR settings were as follows: 50°C for 2 min, then 95°C for 10 min, followed by 40 cycles of 95°C for 15 s, and 60°C for 1 min. HIV-1 DNA/RNA was quantified using standard curve established by ACH-2 cell line that contains one integrated copy of HIV-1 DNA per cell. The detection limit is below 10 HIV-1 DNA copies/10^6^ human CD45+ cells. Human CD45 (Hs0036534_g1) (Life Technology, California, USA) was employed as reference gene for normalization.

### Immunohistochemistry

Hu-HSC mouse tissues were collected at animal autopsy and immediately fixed with 4% paraformaldehyde. After 24 hours, tissues were processed and paraffin embedded. Tissues were cut into 5 μm thick sections and immune-stained with anti-human HLA-DQ/DP/DR (clone CR3/43, 1:100, DAKO, Carpinteria, CA) and HIV-1 p24 (1:10, DAKO, Carpinteria, CA). Images were acquired with a Nuance EX camera fixed to a Nikon Eclipse E800 microscope using Nuance software (Cambridge Research & Instrumentation, Woburn, MA). Images were captured at 10x and 20x magnifications.

### RNAscope

Hu-HSC mouse brain tissues were prepared in 5-μm thick paraffin-embedded sections for RNAScope® analysis (Advanced Cell Diagnostics, Hayward, CA) according to the manufacturer’s instructions. To detect HIV-1 RNA, an anti-sense HIV-1 Clade B probe targeting viral base pairs 854–8291 was employed. Positive signals were detected as single or clusters of brown dots. Images were captured at 40x magnification.

### ddPCR for detection of HIV-1 nucleic acids

Droplet digital PCR (ddPCR) was performed based on the water–oil emulsion droplet technology, using the ddPCR™ Supermix for Probes and reagents in the QX200™ Droplet Digital™ PCR system (Bio-Rad Laboratories, Hercules, CA) (Dash, et al. 2019). For quantification of HIV-1 DNA, the eluted brain DNA was PCR-amplified using Taqman set targeting the HIV-1 gag gene and as a reference human CD45 gene. A total of 400 ng DNA from each tissue was used as template for first round of PCR amplifications with the same thermal cycling conditions used for real-time q-PCR detection. 500ng DNA of the first round semi-tested PCR product was used for the ddPCR reaction, the data was expressed as HIV-1 copies/μg of DNA used. Data acquisition and analysis were done using QX200 droplet reader and QuantaSoft™ software provided with the instrument.

### Statistical Analyses

Data were plotted and analyzed using GraphPad Prism 8.0 software (La Jolla, CA).

## Results

### Donor Hu-HSC Mice

**Figure 1.**
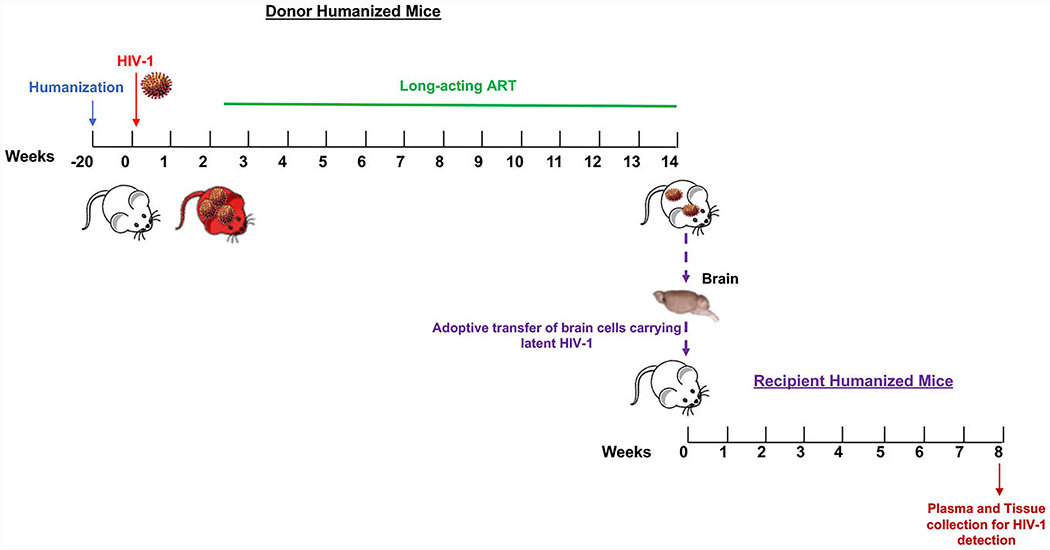
Experimental Scheme. Donor hu-HSC mice were infected with HIV-1_ADA_ intraperitoneally (i.p.) at 10^4^ tissue culture infective dose_50_ (TCID_50_)/ml for 2 weeks before treatment initiation. Plasma viral load (VL) was measured after 2 weeks to detect viral RNA. Suppressive LA-ART was maintained for another 12 weeks before animal sacrifice. Donor hu-HSC mouse brain cells which contained a mixture of mouse and human cells were isolated and immediately intraperitoneally injected into naive hu-HSC mice. Recipient animals were maintained for another 8 weeks before sacrifice and were analyzed for HIV-1 recovery.

**Figure 2.**
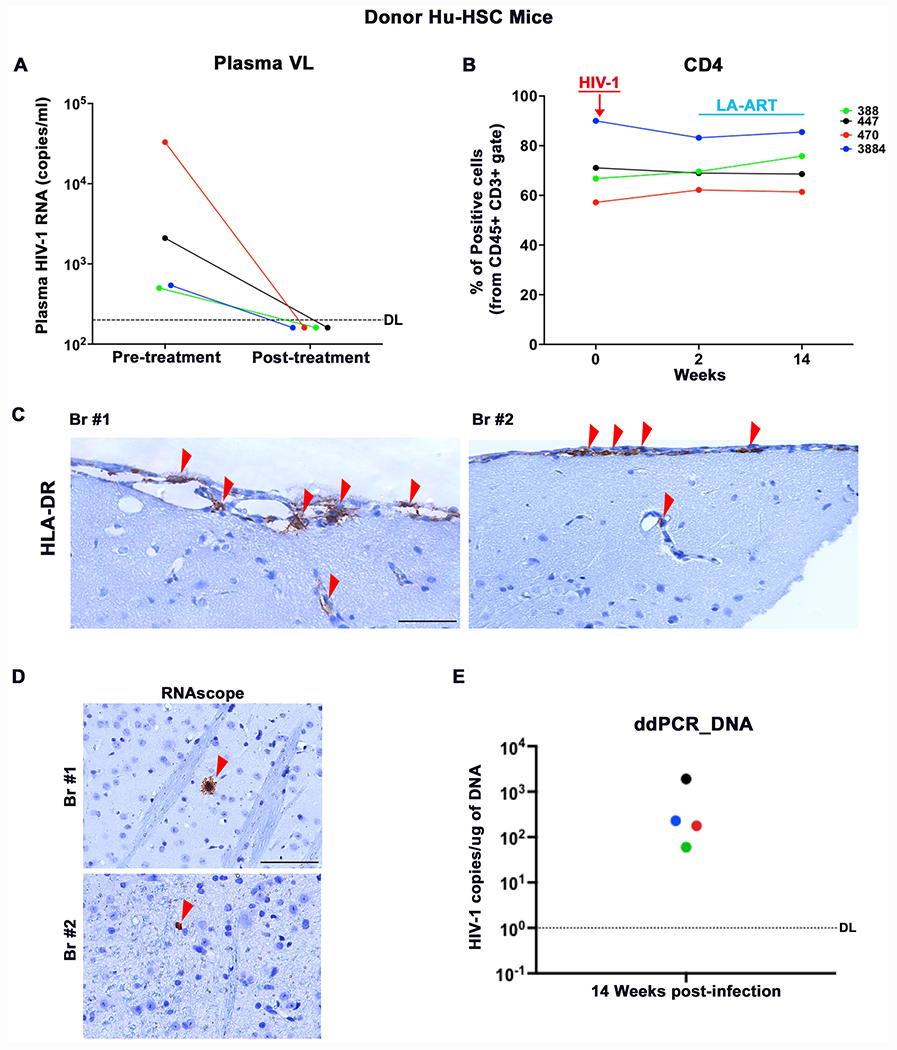
Donor hu-HSC mice. The dynamics of **(A)** plasma viral load (VL) and **(B)** peripheral blood human CD4+ T cells in individual donor hu-HSC mice were recorded. The detection limit (DL) for plasma HIV-1 RNA capture was below 200 copies/ml as shown by the dotted line. Paraffin-embedded brain sections were collected and subjected to **(C)** immunohistochemistry staining for human HLA-DR as well as **(D)** RNAscope analysis for HIV-1 RNA detection as shown by a single or clusters of brown dots. **(E)** Ultrasensitive ddPCR, with sensitivity of detecting 1–2 viral copies, was used in cross validation tests for viral DNA detection from brains.

The experimental scheme used in this study is shown in Figure 1. At 20-weeks of age, donor hu-HSC mice (peripheral human CD45+ cells of 20% or greater) were selected. These mice were intraperitoneally infected with 10^4^ tissue culture infection dose_50_ (TCID_50_)/animal by the intraperitoneal route. Four hu-HSC mice were infected with HIV-1 for 2 weeks before ART was initiated. We chose this timing based on our prior observations that in this humanized mouse model HIV-1 infection is readily established in lymphoid tissues but not yet detected in the brain at 14 days (Su, et al. 2019). At this point, the brain reservoir size is limited while peripheral HIV-1 infection is established. In the donor hu-HSC mice, plasma HIV-1 RNA level ranged from 500 to 33,000 copies/ml before treatment initiation (Figure 2A and Table 1). There were no significant changes in the numbers of peripheral human CD4+ T cells (Figure 2B). Donor hu-HSC mice with affirmed viral infection were administered four long acting ART regimens [LA dolutegravir (NMDTG), rilpivirine (NRPV), lamivudine (NM3TC), and abacavir (NMABC)] (Dash, et al. 2019). All formulations were administered intramuscularly. NMDTG and NRPV were injected once every 4 weeks at 45 mg/kg and NMABC and NM3TC were administered once a week at 50 mg/kg of parent. Drug concentrations reflected parent drug concentrations. ART was initiated at 2 weeks after infection then maintained for 12 weeks before donor hu-HSC mice were sacrificed and adoptive transfer was performed. This treatment scheme induced vigorous HIV-1 suppression in both hu-HSC mouse PBMC and lymphoid and brain tissues (Dash, et al. 2019; Su, et al. 2020). Before animal euthanasia, plasma HIV-1 RNA was confirmed below detection limit (200 copies/ml) in all 4 donors (Figure 2A). Peripheral CD4+ T cells did not show significant changes compared to pre-infection levels (Figure 2B). Peripheral human CD45, CD3, CD19, and CD8 cells all remained unchanged (data not shown). At the conclusion of the experiment, donor hu-HSC mice were sacrificed and animal brains were collected for viral and adoptive cell transfer tests. In the brain sections of donor hu-HSC mice, human cells (stained using anti-human specific HLA-DR) were observed in the meninges and in the cortical regions using immunohistochemistry (Figure 2C). However, at the same view, HIV-1p24 signal was not detected (data not shown). Reflective of our prior (Su, et al. 2020) studies, after 12 weeks of first-generation ART administration, brain HIV-1 infection was restricted where viral RNA and DNA were below the detection limit (10 copies/10^6^ human CD45+ cells). This finding was observed in all the donor hu-HSC mice; measured using semi-nested qRT-PCR (data not shown). RNAscope was deployed for HIV-1 RNA detection. This technique was used with an anti-sense HIV-1 Clade B probe targeting viral base pairs 854–8291. Positive signals were detected as single or clusters of brown dots. By performing RNAscope assay that nearly covers the whole viral sequence (except LTR region) and detects 1~2 viral copies, HIV-1 RNA was captured as sporadic single brown dots in the brain sections of M388 and M3884, but not in M447 and M470 (Figure 2D). Furthermore, ddPCR was used with a sensitivity of 1~2 viral copies for HIV-1 detection. Viral DNA was not detected by direct amplification tests. However, we were successful in capturing low levels of HIV-1 DNA from all donor brains when analyzing 500 ng of DNA products from the first round of semi-nested PCR (Figure 2E). To this end, HIV-1 infection in the brains of donor hu-HSC mice that received early and sustained LA-ART treatment was suppressed whereas viral fragments were detected only by ultrasensitive tests.

### Brain Cell Recipient Hu-HSC Mice

**Figure 3.**
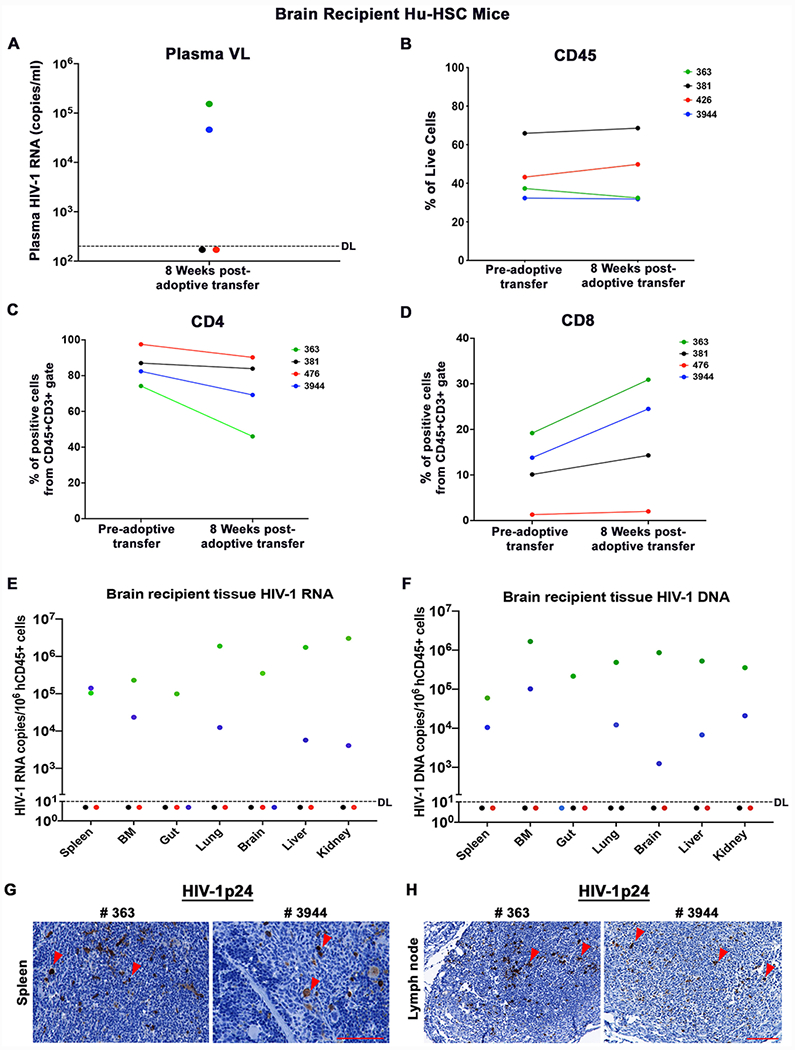
Brain Cell Recipient hu-HSC mice. **(A)** Plasma viral load (VL) after 8 weeks of donor hu-HSC mouse brain engraftment. The detection limit (DL) was below 200 copies/ml as shown by the dotted line. **(B)** Total human cells as stained by flow cytometry using CD45 antibody, at pre-and post-adoptive transfer of brain cells. **(C)** Peripheral CD4+ and **(D)** CD8+ T cells at pre- and post-engraftment. **(E)** Tissue HIV-1 RNA and **(F)** DNA detection in spleen, bone marrow (BM), gut, lung, brain, liver, and kidney by semi-nested real-time qPCR. The DL was below 10 copies/10^6^ human CD45+ cells as shown by the dotted line. Representative **(G)** spleen and **(H)** Lymph node sections from recipient humanized mice (#363 and 3944) showing HIV-1p24 cells as brown color dots by IHC staining. The scale bar indicates 20x and 10x magnifications respectively.

Highly-sensitive PCR based assays only detect partial HIV-1 genomes that do not differentiate replication-defective from replication-competent virus. To investigate whether there was residual replication-competent HIV-1 in virally suppressed donor hu-HSC mouse brains and their capability to induce new round of infection, we resuspended animal brain cells and adoptively transferred them into respective new recipient naive hu-HSC mice through intraperitoneal route (Figure 1 and Table 1). To avoid cell loss during human cell isolation, we engrafted a mixture of mouse and human cells ranging from 52.2~106.4 × 10^6^ cells which included 0.9 ~2.0 x 10^6^ human cells as calculated using flow cytometry analysis (Table 1). Given that HIV-1 rebound usually occurs within 4 weeks upon adoptive transfer (Charlins, et al. 2017; Henrich, et al. 2017; Metcalf Pate, et al. 2015), we maintained recipient hu-HSC mice for 8 weeks to monitor HIV-1 rescue. HIV-1 was successfully recovered from recipient M363 and M3944 but not from M381 and M476 (Figure 3A and Table 1). The total human cells (CD45) remained consistent in all the recipient mice throughout the experimental period as measured using flow cytometry (Figure 3B). There was a decrease of 28.2, 3.1, 7.3, and 13.2% of peripheral human CD4+ cells and an increase of 11.7, 4.2, 0.7, and 10.7% of peripheral CD8+ cell in M363, M381, M476, and M3944, respectively (Figures 3C-D). In the tests used in this report, first round of PCR was performed by conventional PCR in 25 μl of a reaction mix containing 500 ng of template and 50 ng each of both primers directed to the HIV-1 gag region of the virus. Five microliters of the first PCR product were diluted to 10 μl with PCR master mix containing two primers at 0.2 μM each and 0.2 μM TaqMan dual-labeled fluorescent probe for real-time PCR detection. HIV-1 DNA and RNA were quantified. The detection limit was recorded at < 10 HIV-1 DNA copies/10^6^ human CD45+ cells. Tissue HIV-1 infection was readily spread in M363 and M3944 as viral RNA and DNA were detected in spleen, BM, gut, lung, brain, liver, and kidney, but not in M381 and M476 (Figures 3E-F). Recipient humanized mouse spleen and lymph node sections were stained with HLA-DR and HIV-1p24 to trace viral proteins. HIV-1p24+ cells were readily observed in the spleen (Figure 3G) and lymph nodes (Figure 3H) sections of M363 and M3944 as shown in brown dots, but were not detected in M381 and M476 sections. Overall, despite a small size reservoir that was only captured by ddPCR, replication-competent HIV-1 persisted in the brains and was successfully recovered from 50% of donor humanized mice that received early and potent suppressive treatment.

## Discussion

The extent of the HIV-1 tissue reservoir rests on the broad viral distribution within a human host. While routinely viral sampling is performed in peripheral blood, the major site of latent virus is in tissue and is in continuous exchange with blood. Brain is the most inaccessible tissue and can only be studied at autopsy. Although HIV-1 DNA has been identified from brain tissues at post-mortem examination, its replication-competence has not been known (Chaillon, et al. 2020; Lamers, et al. 2016). Simian immunodeficiency virus (SIV)-infected macaque studies have broadened the recognition of latent viral anatomical tissue sites including those in the brain (Gama, et al. 2018; Henderson, et al. 2020). Viral infection is established in the brain within a week after infection and persists for years after suppressive ART (Borducchi, et al. 2018; Graham, et al. 2011). Notably, the primary cell types that harbor latent HIV-1 reservoir in the brain are macrophages and microglial cells (Abreu, et al. 2019; Wallet, et al. 2019). Indeed, using a macrophage QVOA (MΦ-QVOA), SIV was amplified from brain CD11b+ cells in virally suppressed macaque, confirming the presence of replication-competent SIV in CNS (Avalos, et al. 2017). Nevertheless, SIV is different from HIV and as such definitive virus-specific data is required which can be obtained from infected humanized mice. Indeed, by transplanting hu-HSCs into immunodeficient newborn rodents, human immune system can readily be reconstituted which supports HIV-1 replication (Gorantla, et al. 2007; Marsden and Zack 2017; Zhang and Su 2012). Human lymphocytes and macrophages can infiltrate into mouse brains and subject to HIV-1 infection (Gorantla, et al. 2010a). To date, the effect of ART on HIV-1 replication and persistence in the brain of this mouse model has not been investigated. Previously we have conducted ART administration and treatment interruption studies on hu-HSC mice and HIV-1 rebound was observed in all the tissue compartments including brain (Dash, et al. 2019; Su, et al. 2020). However, it was not determined if the reappeared virus came from brain or the peripheral immune system. Recently a humanized mice-based MVOA was developed to recover HIV-1 from aviremic patient PBMCs (Schmitt and Akkina 2018). Notably, MVOA was able to capture low levels of latent viral reservoirs that was not detected by QVOA (Charlins, et al. 2017; Henrich, et al. 2017). We further expanded MVOA using humanized mice as donors and successfully recaptured HIV-1 not only from animal blood leukocytes but also from splenocytes and BM cells (Su, et al. 2020). These observations suggest that hu-MVOA can be used to assay HIV-1 anatomical site and provides an opportunity to investigate brain viral reservoirs.

In this proof-of-concept study ART was administrated to infected humanized mice following the confirmation of viral infection. All infected animals demonstrated pre-treatment viral loads over the detection limit (200 HIV-1 RNA copies/ml) but much lower than SIV infection during a similar infectious window [10^8^ SIV RNA copies/ml, (Avalos, et al. 2017; Barouch, et al. 2016)], suggesting a much smaller pre-treatment brain HIV-1 reservoir size in humanized mice compared to that in macaques. In addition, compared to conventional ART used in the macaque study, LA-ART used in the current study shows superior tissue penetration that may further restrict brain HIV-1 infection (Edagwa, et al. 2017; Sillman, et al. 2018; Zhou, et al. 2018). Together, recovery of brain HIV-1 reservoir from humanized mice has proven more complex based on size and distribution than was reported in rhesus macaques (Avalos, et al. 2017). Indeed, after 12 weeks of suppressive ART treatment HIV-1 RNA and DNA were undetectable in hu-HSC mouse brains by nested qRT-PCR. More sensitive ddPCR captured HIV-1 DNA only by assaying viral sequences from first round product of nested PCR, demonstrating low level of residual HIV-1 in the donor brains. Although HIV-1 RNA was captured in 2 mice brains by sensitive RNAscope assay which detects 1~2 viral copies and covers nearly entire HIV-1 sequence, the single dot signal indicated there was no viral spread. The detected HIV-1 RNA may arise from sporadic amplification of residual virus which could be replication-defective. The successful recovery of HIV-1 dissemination in recipient hu-HSC mice demonstrated the presence of functional viral reservoirs that persisted in the brains despite long-term suppressive ART treatment. Capturing the small reservoir size by hu-MVOA supports it use as a highly sensitive tool for HIV-1 detection. Interestingly, the 2 donors (M388 and M3884) that were captured with brain latent reservoirs had barely detectable pre-treatment plasma HIV-1 RNA (500 and 540 copies/ml) indicating that brain HIV-1 reservoirs were established during very early stages of infection even when the peripheral viral level was low. The failure of restoring HIV-1 amplification from 2 other donors (M447 and M470) likely resulted from small reservoir size in the engrafted cells, which was also observed in previous reports (Charlins, et al. 2017; Henrich, et al. 2017). Lower numbers of engrafted human cells from the negative donors (0.9 and 1.1 million) compared to the positive donors (1.7 and 2.0 million) may also contribute to unsuccessful viral recovery. Another explanation for the absence of detectable viral reservoirs could be the focal distribution of HIV-1 infection in the brain as reported by other researchers (Avalos, et al. 2017; Gama, et al. 2017). Adoptively transferring different compartments of brain tissues may provide more insights. The limitation of current study is lack of sensitivity comparison between QVOA and MVOA on the detection of brain reservoirs due to limited reconstitution of human cells in hu-HSC mouse brains (Gorantla, et al. 2012). It can be realized by the development of novel humanized mouse models with improved human cell reconstitution in the rodent brains (Mathews, et al. 2019).

In conclusion, we posit that this humanized mouse-to-mouse MVOA (hu-MVOA) can be used to assess HIV-1 infection in the CNS. Replication-competent HIV-1 can be established in brain during long-term ART suppressive treatment. The data support further investigations on the use of hu-MOVA to interrogate tissue viral reservoirs. Hu-MVOA extended humanized mice as a valuable pre-clinical model to study HIV-1 persistence and could be an innovative system in studies of viral eradication.

## Figures and Tables

**Table 1.** Descriptors of donor and recipient humanized mice

Donor hu-HSC mice						Recipient hu-HSC mice		
Animal ID	Plasma HIV-1 RNA (copies/ml) LA-ART	Plasma HIV-1 RNA before AT (copies/ml)	Peripheral hCD45 before AT (%)	No. of total viable brain cells for AT(x10_6_)	No. of total viable human cells for AT (x10_6_)	Animal ID	Peripheral hCD45 before AT(%)	Plasma HIV-1 RNA 8 weeks after AT (copies/ml)
388	500	ND	58.7	105.4	2.0	363	37.3	153000
447	2100	ND	30.6	108.4	1.1	381	75.9	ND
470	33000	ND	34.0	52.2	0.9	476	43.2	ND
3884	540	ND	29.5	54.8	1.7	3944	27.3	46000

# AT, adoptive transfer; ND, not detected < 200 copies/ml.

## Data Availability

All the data findings from this study are included with this manuscript, any other relevant data supporting the key findings of this study are available from the corresponding authors upon reasonable request.
